# Th17 cells with regulatory phenotype are the main IL-17F and IL-26 producers in palmoplantar pustulosis

**DOI:** 10.1172/jci.insight.193038

**Published:** 2025-09-23

**Authors:** Tran H. Do, Rachael Bogle, Haihan Zhang, Xianying Xing, Mehrnaz Gharaee-Kermani, Madalina Raducu, Jennifer Fox, Rundong Jiang, Olesya Plazyo, Paul W. Harms, Mio Nakamura, Enze Xing, Michel Gilliet, Allison C. Billi, J. Michelle Kahlenberg, Robert L. Modlin, Ozge Uluckan, Lam C. Tsoi, Johann E. Gudjonsson

**Affiliations:** 1 Department of Dermatology, University of Michigan Medical School, Ann Arbor, Michigan, USA.; 2 Almirall SA, R&D Center, Sant Feliu de Llobregat, Barcelona, Spain.; 3 Department of Pathology, University of Michigan Medical School, Ann Arbor, Michigan, USA.; 4 Department of Dermatology and Venereology, University Hospital of Lausanne, Lausanne, Switzerland.; 5 Department of Internal Medicine, Division of Rheumatology, University of Michigan, Ann Arbor, Michigan, USA.; 6 A. Alfred Taubman Medical Research Institute, Ann Arbor, Michigan, USA.; 7 Division of Dermatology, UCLA, Los Angeles, California, USA.

**Keywords:** Dermatology, Immunology, T cells

## Abstract

Palmoplantar pustulosis (PPP) is a chronic inflammatory skin disorder marked by erythematous pustules and desquamation on the palms and soles. While IL-17 pathways are implicated in PPP, IL-17 blockers have shown modest efficacy, underscoring the need for a deeper understanding of IL-17 involvement. To dissect the cellular and spatial architecture of PPP, we performed single-cell RNA-Seq (scRNA-Seq) on lesional, nonlesional, and healthy acral skin to examine cellular composition, transcriptomic profiles, and cell-cell interactions. Unbiased clustering revealed 9 major cell types, including an inflammatory keratinocyte subset enriched in IL-17A/TNF signatures and marked by high IL-36G expression. Within the lymphocyte compartment, we identified a hybrid “regTh17” population coexpressing regulatory markers (*FOXP3*, *CTLA4*, *TIGIT*), *IL17F*, and *IL26*. This regTh17 subset was distinguished by elevated *IL1R1* and *CD39*, suggesting an IL-1β–driven differentiation. Spatial analyses demonstrated significant neighborhood enrichment of regTh17 cells with IL-36G^+^ supraspinous keratinocytes. RegTh17 cells were the predominant source of IL-17F and IL-26 signals, whereas keratinocytes were predicted as their main receivers. We further observed regTh17 coexpressing TNFRSF4 (OX40) and TNFRSF18 (GITR) specifically at sites of IL36G^+^ keratinocyte interactions, implicating these pathways in amplification of the IL-17/IL-36 inflammatory loop. Together, our integrated single-cell and spatial profiling uncovers Th17 plasticity in PPP, identifies a regTh17-keratinocyte interaction, and highlights IL-17F, IL-26, OX40/OX40L, and GITR/GITRL as candidate targets for precision therapies in this challenging disease.

## Abstract 2

A unique subset of Th17 cells with regulatory phenotype drives inflammation in palmoplantar pustulosis by producing IL-17F and IL-26 and interacting with inflammatory keratinocytes.

## Graphical abstract

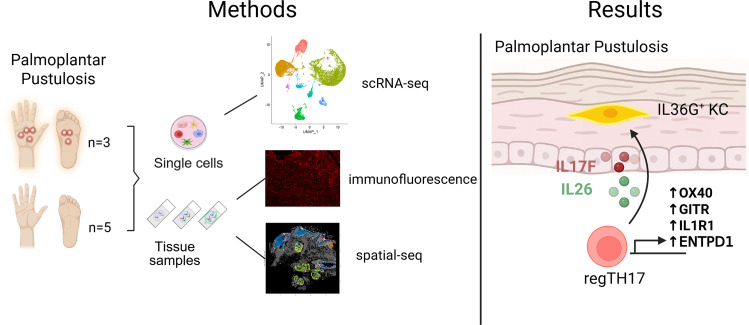

## Introduction

Palmoplantar pustulosis (PPP) is a chronic inflammatory skin disease that is often considered a variant of pustular psoriasis. PPP presents with sterile pustules affecting the palms of the hands and the soles of the feet (1). In severe cases, the disease progresses to large erythematous, painful plaques covering the entire surface of palms and soles. While the prevalence of PPP in the United States ranges from 0.028% to 0.12%, PPP is associated with greater impairment in quality of life compared with other types of psoriasis due to its painful and disabling nature (2–6). While studies have shown involvement of IL-17 pathways in the pathogenesis of PPP (7, 8), clinical trials investigating IL-17 blocking antibodies for the treatment of PPP have yielded conflicting results (9–11). The genetic and immune etiology of PPP is multifaceted and not fully understood (7, 8, 12–15). Key cytokines such as TNF and IL-23 have been implicated in driving the inflammatory cascade in PPP (7, 8, 16). Additionally, overexpression of IL-36 is thought to contribute to the autoinflammatory aspects of the disease, promoting keratinocyte proliferation, neutrophil infiltration, and pustule formation (12).

Advances in single-cell RNA-Seq (scRNA-Seq) have provided new tools to investigate the cellular landscape and the cell-signaling dynamics and have accelerated the understanding of the pathogenic players behind various inflammatory skin diseases. Here, we applied scRNA-Seq to lesional and nonlesional palmoplantar skin from patients with PPP alongside acral samples from healthy donors, uncovering a previously unrecognized Th17 subset that coexpresses canonical regulatory markers yet drives robust *IL17F* and *IL26* expression. To place these findings in tissue context, we then employed spatial transcriptomics, which mapped this regulatory Th17 (regTh17) population into the supraspinous epidermal compartment and revealed its close apposition to IL-36G^+^ keratinocytes. By integrating single-cell and spatial data, our study defines a cellular circuit underpinning PPP inflammation and highlights new targets for therapeutic intervention.

## Results

**Figure 1:**
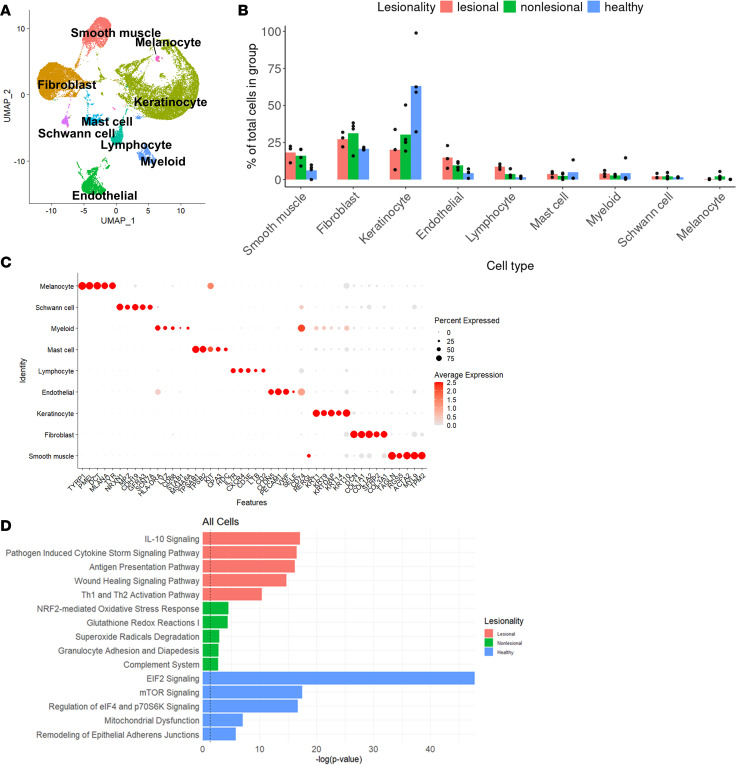
Cellular composition of palmoplantar pustulosis skin and healthy skin. (**A**) Uniform manifold approximation and projection (UMAP) of 32,364 cells from lesional and nonlesional skin biopsies of 3 patients with PPP and 5 healthy controls colored by cell types. Ingenuity pathway enrichment significance was determined using a right-tailed Fisher’s exact test, and results are displayed as –log(p-value). (**B**) Bar plot depicting the proportional distribution of cell types across disease conditions (lesional, nonlesional, and healthy), with individual donor proportions represented as dots. (**C**) Dot plots illustrating selected marker genes expression for cell types. (**D**) Enriched IPA across lesional (red), nonlesional (green), and healthy (blue) with significance indicated by the dotted line (–log[*P* value]).

To understand the cellular heterogeneity of PPP, we performed scRNA-Seq on lesional and nonlesional skin biopsies from 3 patients with PPP and acral skin from 5 healthy controls (Table 1). After quality control, we identified 32,364 cells with an average of 2,419 genes and 10,056 transcripts detected per cell. Using unbiased clustering methods from Seurat R package, we identified 25 clusters corresponding to 9 major cell types (Figure 1, A and B). All 9 major cell types were present in all 3 PPP samples (Supplemental Figure 1A; supplemental material available online with this article; https://doi.org/10.1172/jci.insight.193038DS1). Major cell types were annotated using known canonical markers (Figure 1C). Lesional skin from PPP had the largest proportion of endothelial cells and lymphocytes compared with nonlesional and healthy controls (Figure 1B). Meanwhile, nonlesional skin had the largest proportion of melanocytes, and healthy controls had the largest proportion of the keratinocytes (KC) (Figure 1B and Supplemental Figure 1B). Ingenuity Pathway Analysis (IPA) of top canonical pathways of PPP lesional-specific genes revealed immune-related pathways such as IL-10 signaling, antigen presentation, and Th1 and Th2 activation, while the nonlesional-specific and healthy skin-specific genes revealed nonspecific cellular processes such as NRF2-mediated oxidative stress response, superoxide radicals degradation, EIF2 signaling, mTOR signaling, and mitochondrial dysfunction (Figure 1D). We also performed cell-cell interaction analysis using CellChat and found that lesional samples exhibited robust interactions of lymphocyte and myeloid cells (Supplemental Figure 1C). In contrast, the cell-cell interactions in nonlesional and healthy samples were highest between lymphocytes and stromal cells like smooth muscle and melanocytes (Supplemental Figure 1C).

**Figure 2:**
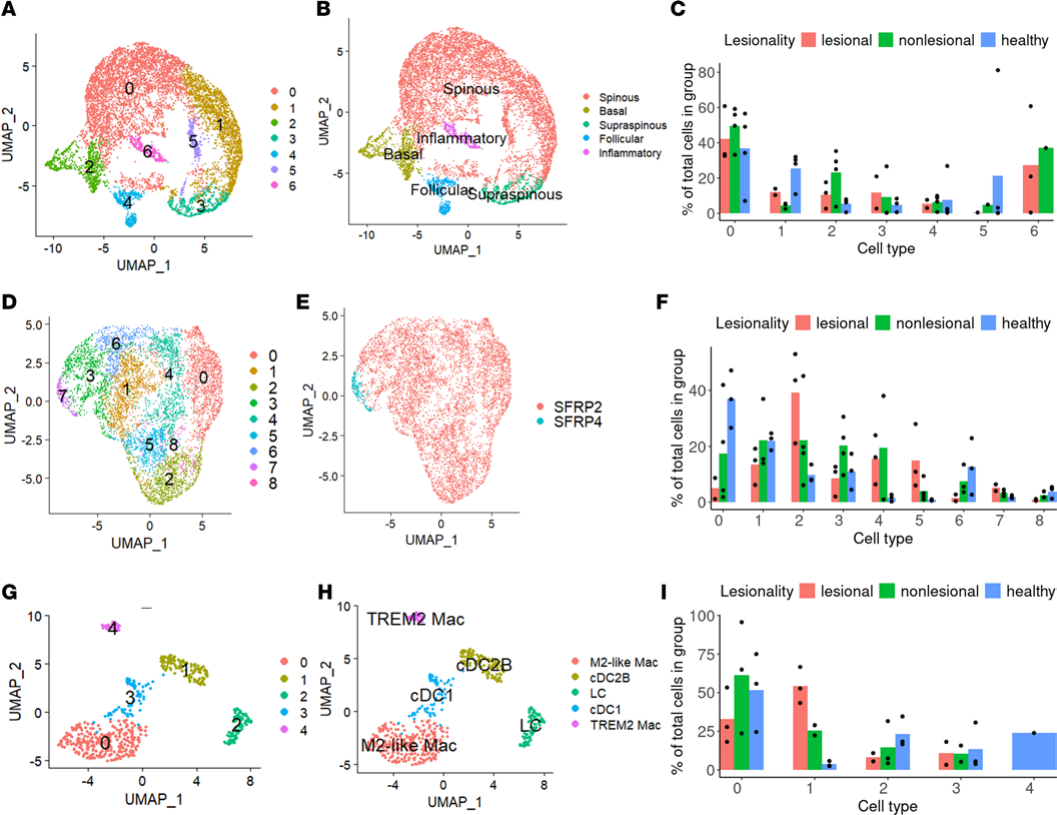
Cellular composition in PPP and healthy skin by subclusters. (**A**) UMAP of 7 subclusters of keratinocytes colored by subclusters. (**B**) UMAP of keratinocytes colored by cell types. (**C**) Bar plot depicting the proportional distribution of keratinocyte subclusters normalized by disease conditions (lesional, nonlesional, and healthy), with individual donor proportions represented as dots. (**D**) UMAP of 9 subclusters of fibroblasts colored by subclusters. (**E**) UMAP of fibroblasts colored by cell types. (**F**) Bar plot depicting the proportional distribution of fibroblast subclusters normalized by disease conditions (lesional, nonlesional, and healthy), with individual donor proportions represented as dots. (**G**) UMAP of 5 subclusters of myeloid colored by subclusters. (**H**) UMAP of myeloid colored by cell types. (**I**) Bar plot depicting the proportional distribution of myeloid subclusters normalized by disease conditions (lesional, nonlesional, and healthy), with individual donor proportions represented as dots.

After stringent quality control filtering and clustering analysis, we identified 7 subclusters of keratinocytes (Figure 2A). Using canonical markers, we annotated 5 subclusters of keratinocytes with *S100A7*, *S100A8*, and *KRT6A* as inflammatory; *KIT*, *KRT8*, and *CD200* as follicular; *LOR*, *FLG*, and *SLURP1* as supraspinous; *KRT5* and *KRT14* as basal; and *KRT1* and *KRT10* as spinous keratinocytes (Figure 2B and Supplemental Figure 2A). Keratinocyte subcluster 6, annotated as inflammatory keratinocytes, was exclusive to the PPP skin (Figure 2C and Supplemental Figure 2, B and C). To understand how each keratinocyte subcluster is regulated, we calculated module scores using genes induced in cultured keratinocytes stimulated by various inflammatory cytokines as previously described (17, 18). Keratinocyte subcluster 6, annotated as inflammatory KC, exhibited the highest IL-17A, TNF, and IL-17–TNF module scores, while keratinocyte subcluster 3, annotated as supraspinous, had the highest IL-36G and IFN-γ module scores (Supplemental Figure 2D), likely a similar population to our previous findings of IL-36G^+^ supraspinous keratinocytes in psoriasis (18).

We analyzed 9 subclusters within fibroblasts (FBs) identifying 2 major FB subclusters, SFRP2 and SFRP4 FB (Figure 2, D and E). We found FB subclusters 4 and 5, which are almost exclusive to PPP skin, had high expression of SFRP2 (Figure 2F and Supplemental Figure 3A). FBs engaged in inflammatory responses in lesional skin as determined by cell-cell interaction strength (Supplemental Figure 1C), suggesting an active role in PPP pathogenesis.

**Figure 3:**
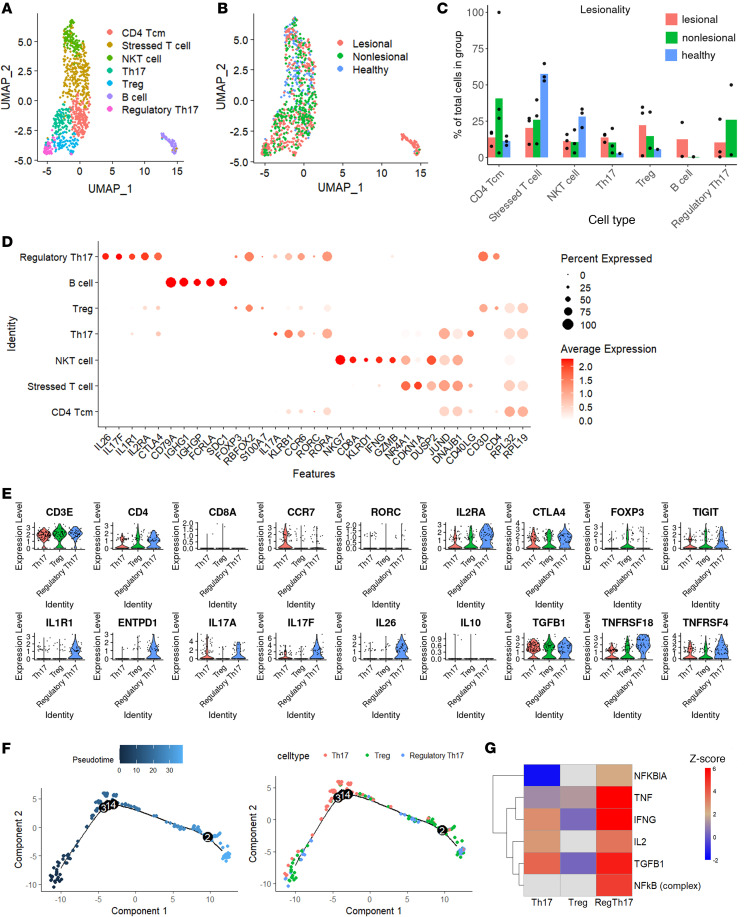
Regulatory Th17 cells identified as a key modulator in the PPP lesional skin. (**A**) UMAP of lymphocytes colored by cell types. (**B**) UMAP of lymphocytes colored by lesional (red), nonlesional (green), and healthy (blue). (**C**) Bar plot depicting the proportional distribution of lymphocyte subclusters normalized by disease conditions (lesional, nonlesional, and healthy), with individual donor proportions represented as dots. (**D**) Dot plot of canonical marker genes for each annotated lymphocyte subclusters. (**E**) Violin plots showing selected genes across Th17, Treg, and regulatory Th17 subclusters. (**F**) Pseudotime trajectory showing the distribution of Th17, Treg, and regulatory Th17 subclusters. (**G**) Heatmap showing the *z* score of IPA upstream regulator for the Th17, Treg, and regulatory Th17 subclusters.

We then examined the immune cell landscape in the dataset. In total, 1,496 immune cells were further analyzed and identified as 894 lymphocytes and 602 myeloid cells based on canonical markers (Figure 1C). For the myeloid compartment, we annotated 5 myeloid types with *CD163*, *LILRB5*, and *STAB1* as M2-like macrophage; *CD1A* and *CD207* as Langerhans (LC); *CD1C*, *CLEC9A*, and *CST3* for cDC1; *CD1C* and *CLEC10A* as cDC2B; and *TREM2*, *APOE*, *APOC1*, and *GPNMB* as TREM2 macrophage (Figure 2, G and H, and Supplemental Figure 3B). Interestingly, the cDC2B subcluster was almost exclusive to PPP skin, while the TREM2 macrophage subcluster was exclusive to healthy skin (Figure 2I). Among lymphocyte populations, B cells were enriched among lesional samples, although only in 1 out of 3 patients (Figure 3, A–C, and Supplemental Figure 4A)

Using canonical marker genes, we identified and annotated 6 distinct T cell clusters. First, CD4^+^ central memory T cells (Tcm) were defined by expression of *CD40LG*, *CD4*, and *RPL32*. A separate cluster of T cells was annotated as “stressed T cell” since it exhibited high levels of stress-inducible genes *NR4A1* (19), *CDKN1A* (20), *JUNB* (21), and *DNAJB1* (22). NK T cells (NKT) coexpressed NK cell markers such as *NKG7* alongside T cell markers *CD8A* and *KLRD1* (19–22). Two clusters represented classical Th subsets: classic Th17 cells, which expressed *CD4*, *RORC*, and *IL17A* but low levels of *IL17F*; and Treg, marked by *FOXP3* and *CTLA4*. Intriguingly, we also discovered a hybrid regTh17 cluster expressing both immunosuppressive markers (*FOXP3*, *CTLA4*, and *TIGIT*) and Th17 cytokines (*IL17A* and *IL17F*) (Figure 3, D and E). This regTh17 population was distinguished by high *IL1R1* expression, linking IL-1β signaling to Th17 differentiation and RORγt stabilization (23) as well as robust *ENTPD1* (CD39) expression, which degrades extracellular ATP to AMP to limit tissue inflammation (24–26). The markedly higher IL-26 production by our regTh17 cells, compared with classic Th17 or Treg population, likely functions as a driver for *IL-17A* and *IL-17F* expression while dampening *IL10* and *IFNG* expression (Supplemental Figure 4B).

The presence of regTh17 observed in all 3 patients with PPP, most prominently in 1 donor, underscores their presence in lesional skin (Supplemental Figure 4, A and C). These 3 populations were exclusive to the PPP samples, with lesional cells making up 60% of the subcluster (Figure 3C).Gene set enrichment analysis revealed that both Th17 and regTh17 cells are strongly associated with proinflammatory and cytokine signaling pathways (e.g., regulation of IL-2 and IL-12 production). However, only regTh17 cells demonstrated enrichment in immunomodulatory programs, such as negative regulation of programmed cell death, and of macromolecule biosynthesis (Supplemental Figure 4D). Pseudotime mapping of Treg, Th17, and regTh17 populations showed extensive intermixing along a continuum rather than discrete end states, suggesting a transitional state rather than a terminal lineage (Figure 3F). Th17 cells bifurcate into a cytokine-driven arm (high *CD28*, *IL17A*, *CD40LG*, *IL22*, *TGFBR2*) and a migratory arm (high *ACTB*, *CTSC*, *CORO1A*) (Supplemental Figure 4E), whereas upstream-regulator analysis revealed *TNF*, *IFNG*, *IL2*, *TGFB1*, and *NFKB* as key drivers of the regTh17 cluster (Figure 3G). Downstream targets OX40 (*TNFRSF4*) and GITR (*TNFRSF18*) are highly expressed in regTh17 cells — both in single-cell data (Figure 3E and Supplemental Figure 4F) and by IHC in PPP lesions (Supplemental Figure 4G). In the lesional PPP skin, we confirmed the high expression levels of IL-26 and IL-17A and their coexpression with FOXP3 (Supplemental Figure 5A). We also verified increased expression of *FOXP3* in the PPP lesional sample by IHC (Supplemental Figure 5B). These findings suggest that regTh17 cells are characterized by a potent inflammatory signature, driven by upstream regulator such as TNF, IFN-γ, and IL-2, while concurrently exhibiting high expression of *IL26*, *IL17A*, and *IL17F* along with upregulation of regulatory pathways that restrain cell death and biosynthesis. This positions regTh17 cells as a hybrid subset that may both amplify local inflammation and possibly limit excessive tissue damage in PPP.

**Figure 4:**
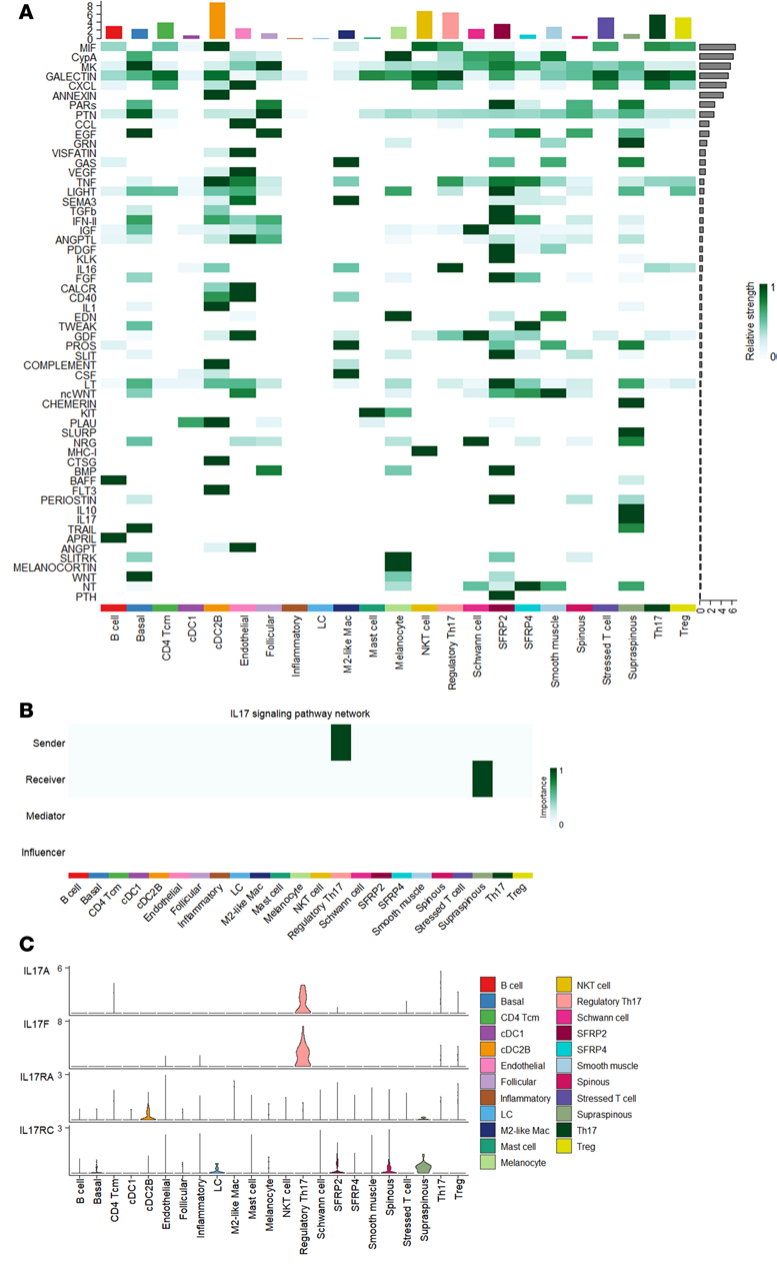
Cell-cell communication analysis revealed IL-17 signaling exclusively in lesional PPP skin. (**A**) Heatmap for outgoing signals associated with each cell type for PPP lesional samples. The top-colored bar plot shows the total signaling strength of a cell group by summarizing all signaling pathways. The right gray bar plot shows the total signaling strength of a signaling pathway by summarizing all cell groups. (**B**) Heatmap of the inferred IL-17 signaling pathway in each cell type for PPP lesional samples. (**C**) Violin plots of genes showing expression levels contributing to the IL-17 signaling pathway in each cell type in PPP lesional samples.

**Figure 5:**
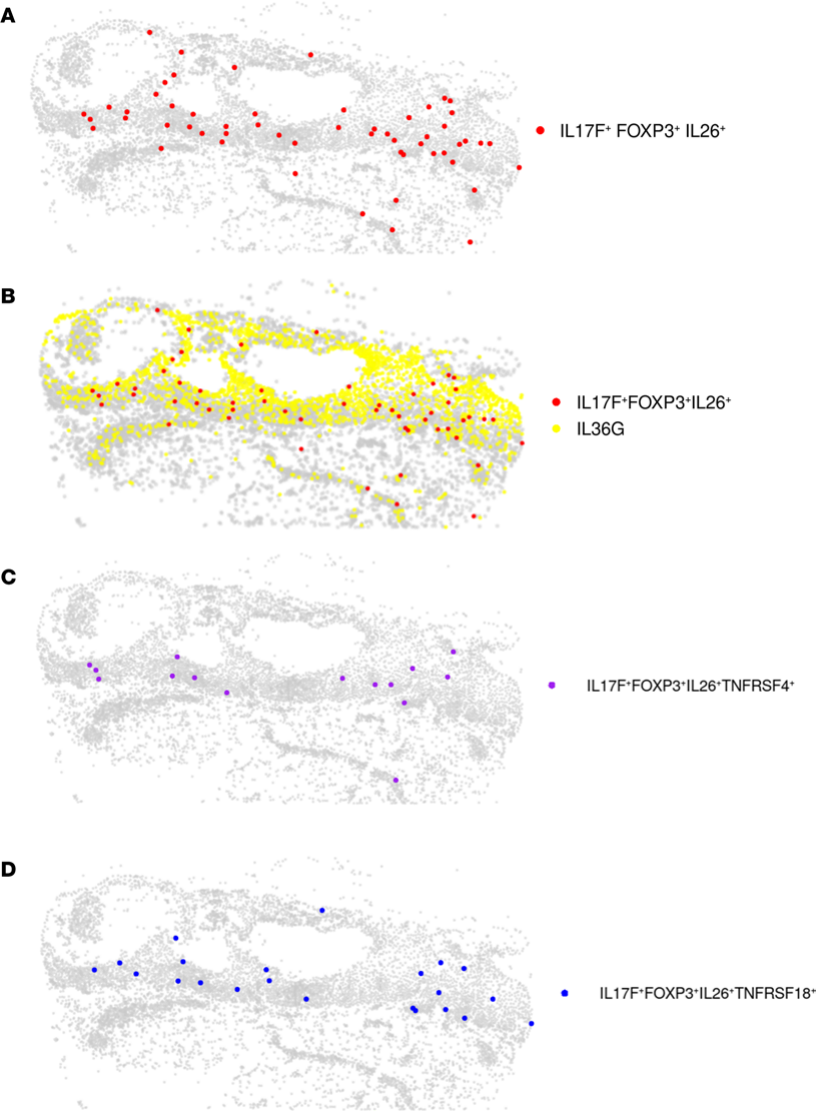
CosMX spatial analysis confirmed regulatory Th17 cells as a key driver of inflammation in PPP skin. (**A**) Spatial transcriptome image of lesional sample showing cells that coexpressed *IL17F*, *FOXP3*, and *IL26* (red). (**B**) Spatial transcriptome image of lesional sample showing cells that coexpressed *IL17F*, *FOXP3*, and *IL26* (red) and IL-36G^+^ cells (yellow). (**C** and **D**) Spatial transcriptome image of lesional sample showing cells that coexpressed *IL17F*, *FOXP3*, *IL26*, and *TNFRSF4* (purple) and *IL17F*, *FOXP3*, *IL26*, and *TNFRSF18* (blue).

Cell-cell communication analysis of outgoing signals on the annotated cell types showed the IL-17 signaling pathway exclusively in lesional PPP skin (Figure 4A and Supplemental Figure 6A). Within this network, regTh17 cells emerged as the predominant senders of IL-17 signals, while supraspinous keratinocytes served as the main receivers (Figure 4, B and C). Notably, keratinocyte subcluster 3, which was annotated as supraspinous KC, displayed the highest IL-36G module score, as we had previously identified (Supplemental Figure 2D) (18). To map regTh17 cells in the skin, we applied the NanoString CosMx 1000-plex panel to lesional PPP samples and used a matched scRNA-Seq reference to compute average signature profiles for each annotated cell type (Supplemental Figure 6B), with corresponding H&E-stained tissue morphology shown in Supplemental Figure 6C. Cell types were localized based on their marker genes (Supplemental Figure 6, B and D). Neighborhood enrichment analysis revealed a spatial association between regTh17 cells and keratinocytes, prompting us to interrogate keratinocyte-derived IL-36G expression within these niches (Supplemental Figure 6E). Individual gene expression patterns for *CD3E*, *CD4*, *CTLA4*, *FOXP3*, *IL17A*, *IL17F*, and *IL26* were plotted to verify the presence regTh17 cells (Supplemental Figure 6, F–M). We confirmed that regTh17 cells, defined by coexpression of *IL17F*, *FOXP3*, and *IL26*, are present lesional skin (Figure 5A). We also validated strong IL-36G expression in supraspinous keratinocytes and demonstrated their close spatial proximity to IL-17F^+^FOXP3^+^IL-26^+^ cells (Figure 5B), mirroring our previous findings on IL-36–dependent amplification of IL-17A and TNF inflammatory responses in IL-36G^+^ keratinocytes (18). The spatial proximity of Treg (*CD4*, *FOXP3*, *IL2RA*), Th17 (*CD4*, *IL17A*, *RORC*), regTh17 (*IL17F*, *IL26*, *FOXP3*), and NKT (*CD8A*, *NKG7*, *KLRD1*) cells to IL-36G^+^ keratinocytes were analyzed, revealing that regTh17 cells had the highest proportion located within 20 μm of IL-36G^+^ cells (Supplemental Figure 6N). Finally, we observed coexpression of TNFRSF18 (GITR) and TNFRSF4 (OX40) at sites where regTh17 and IL-36^+^ keratinocytes interact (Figure 5, C and D). These costimulatory interactions may further amplify the inflammatory cascade, implicating OX40L/OX40 and GITR/GITRL signaling in the pathogenesis of PPP.

## Discussion

Our scRNA-Seq analysis of PPP lesional skin biopsies, compared with PPP nonlesional skin and healthy controls, has provided significant insights into the cellular heterogeneity and immune landscape of PPP. Unlike generalized pustular psoriasis, which affects widespread areas of the body, PPP is confined to the palms and soles (1). This localization suggests that PPP may have unique pathogenic mechanisms and environmental triggers. Despite being grouped under pustular psoriasis, the exact pathogenesis of PPP remains ill defined, complicating efforts to delineate it clearly from other forms of psoriasis (27). The recalcitrant nature of the disease to treatments such as anti–IL-17 treatments further underscore the need for more targeted therapeutic strategies specific to PPP.

The acral skin of the palms and soles has a unique immunological environment that may contribute to the distinct presentation of PPP. Acral skin is thicker with a unique lipid and immune gene expression compared with other body sites (27). These factors create a specialized niche influencing the local immune response and driving treatment response. The analysis revealed notable differences in cellular compositions among lesional PPP, nonlesional skin, and healthy controls. Lesional PPP skin exhibited a higher proportion of endothelial cells and lymphocytes, suggesting an enhanced immune and vascular response. In addition, cDC2B myeloid cells and antigen presentation pathway were found to be enriched in PPP skin, suggesting an underlying antigen-driven process.

T cell plasticity, particularly the dynamic interplay between Th17 cells and Treg, seems to be characteristic to PPP pathogenesis, and has also been described in the blood of patients with PPP where a Th17/Th2 plasticity was observed (28). Th17 cells, which produce cytokines such as IL-17A, have previously been suggested as key drivers of inflammation in PPP (7). Within the keratinocytes, we defined 5 functional subclusters and identified an inflammatory keratinocyte cluster exclusive to PPP lesions. These cells exhibited the highest IL-17A, TNF, and IL-17–TNF module scores, implicating cytokine-driven keratinocyte activation in disease perpetuation. Supraspinous keratinocytes, by contrast, were enriched for IL-36G and IFN-γ signatures, aligning with their spatial proximity to IL-36–mediated inflammatory loops.

A major finding is the discovery of a hybrid regTh17 subpopulation coexpressing immunosuppressive markers (*FOXP3*, *CTLA4*, *TIGIT*) alongside proinflammatory cytokines (*IL17A*, *IL17F*, *IL26*). These cells were enriched for *IL1R1* and *ENTPD1* (CD39), linking IL-1β signaling and extracellular ATP hydrolysis to Th17 differentiation and immune regulation. The high IL-26 output by regTh17 cells may further amplify IL-17 production while restraining IL-10 and IFN-γ, suggesting a self-reinforcing inflammatory loop distinct from classic Th17 or Treg biology.

Pseudotime analysis revealed that regTh17 cells occupy a transitional state, sharing transcriptional programs with both Treg and Th17s, and bifurcating into cytokine-driven and migratory trajectories under the influence of TGFB1, IL-2, and TNF signaling, as previously reported and proposed to be driven largely by TGF-β and IL-6 (29–31). Interestingly, IL-1R1 was more prominently expressed in the regTh17 cells than in classic Th17 cells or Treg. IL-1 signaling has been implicated in the development of human Th17 cells (23) and, in particular, differentiation of Th17 from FOXP3^+^ naive Treg (32). In our data, the expression of this receptor is accompanied by a shift toward higher IL-17F/IL-26 expression. Thus, this shift toward IL-17F at the cost of lower IL-17A could explain the relatively modest response rates seen with narrow IL-17A targeting in PPP (33).

Upstream-regulator analysis implicated TNF, IFN-γ, IL-2, TGFβ1, and NF-κB as key drivers of regTh17 differentiation, with downstream targets OX40 (TNFRSF4) and GITR (TNFRSF18) highly expressed in these cells. Spatial mapping via NanoString CosMx confirmed the close apposition of regTh17 cells and IL-36G^+^ supraspinous keratinocytes, as well as coexpression of OX40 and GITR with regTh17 at the site of the regTh17-keratinocyte interactions, underscoring their role in amplifying local inflammation. These interactions represent key amplifiers of the local immune response. OX40 (TNFRSF4) signaling through its ligand OX40L (TNFSF4) is well documented to enhance the survival and cytokine production of effector T cells, while GITR (TNFRSF18) signaling is known to modulate Treg function and promote effector T cell activity (34). In the context of PPP, the interaction of these pathways between regTh17 cells and IL-36G^+^ KC likely contributes to the robust inflammatory response and chronicity of the disease. These findings provide evidence for how OX40/OX40L and GITR/GITRL signaling may synergize with IL-17 and IL-36 signaling networks, amplifying the inflammatory loop within lesional PPP.

While our study demonstrated that the majority of IL-26 was expressed in this regTh17 subpopulation, recent research has highlighted neutrophils as the main producer of IL-26 in PPP (35). The study found that human neutrophils drive skin autoinflammation by producing IL-26, which promotes the activation and proliferation of Th17 cells and keratinocytes (35). Our study was not able to identify neutrophil subclusters, given the difficulty of isolating neutrophils by scRNA-Seq from fresh skin tissue biopsies. Despite these differences, both studies underscore the critical role of IL-26 in the pathogenesis of PPP. This convergence of findings highlights IL-26 as a pivotal cytokine in the inflammatory network of PPP, potentially serving as a valuable therapeutic target.

While recent studies have provided valuable insights into the cellular and molecular mechanisms of PPP, there are several limitations. Much of the current work is descriptive or correlative, lacking the mechanistic depth required to establish causative relationships. However, the advent of scRNA-Seq and spatial transcriptomics has generated rich datasets that offer unprecedented detail about the cellular composition and spatial organization of PPP lesions. We identified key cellular players, such as regTh17 cells and their interactions, laying the groundwork for more mechanistic studies. Future research should aim to leverage these datasets to perform functional analyses and validate potential therapeutic targets in PPP.

A limitation of our study is that control samples were predominantly palmar skin, whereas PPP lesional samples were from the soles. Although key cell types were observed across both sites (Supplemental Figure 1A), we cannot fully exclude site-specific effects contributing to the differences we report. Notably, FOXP3^+^ Treg and CD8^+^ T cells were not recovered from our healthy skin controls. The overall frequency of skin-resident Treg and CD8^+^ cells in noninflamed skin is low, and our previous findings have indicated that acral skin sites have differences in innate and adaptive immune response compared with nonacral skin (36). In addition, we cannot exclude the possibility that the increased thickness of the epidermal layer may have contributed to overall “dilution” of dermal immune subsets. While our CosMx spatial profiling provided valuable proof-of-principle insights into regTh17-keratinocyte interactions in lesional PPP skin, these data derive from a single sample and must therefore be considered preliminary. The lack of additional PPP precludes formal statistical comparison and leaves open the possibility of interindividual or technical batch effects. Future studies should expand the CosMx cohort — and ideally include orthogonal validation (e.g., multiplex IF or RNAscope across multiple patients) — to confirm the spatial patterns and improve the generalizability of our findings.

Our comprehensive scRNA-Seq and spatial analysis of PPP skin biopsies have revealed insights into the cellular and molecular landscape of this complex disease. The unique immunological environment of acral skin, characterized by its specialized structure and immune gene expression, plays a critical role in shaping the local immune response and the pathogenesis of PPP. Among the key findings, we identified a regTh17 subpopulation as a potential contributor to PPP pathology, characterized by coexpression of *IL17F*, *IL26*, and *FOXP3*. These cells not only exhibit features of both Th17 and Treg but also engage in direct interactions and in close proximity with inflammatory IL-36G^+^ supraspinous keratinocytes.

Our findings further highlight the significance of OX40/OX40L (TNFRSF4/TNFSF4) and GITR/GITRL (TNFRSF18/TNFSF18) signaling pathways in amplifying the inflammatory network within PPP lesions. These interactions between regTh17 cells and keratinocytes likely contribute to the chronicity and robustness of the inflammatory response, suggesting a central role for these pathways in PPP pathogenesis. Together with the IL-17 and IL-36 signaling axes, these pathways form a tightly interconnected inflammatory loop that perpetuates local immune activation and keratinocyte dysfunction.

By elucidating these pathways, our study provides a foundation for future research into PPP-specific mechanisms and therapeutic targets. Targeting molecules, such as IL-17F, IL-26, OX40/OX40L, and GITR/GITRL, may offer new avenues for intervention in this refractory disease. While further mechanistic studies are necessary to validate these targets and explore their potential for clinical translation, this work underscores the importance of integrating single-cell and spatial transcriptomic approaches to unravel the complexities of skin inflammation and highlights the need for personalized, site-specific therapeutic strategies for diseases like PPP.

## Methods

### Sex as a biological variable.

Sex was not considered as a biological variable.

### Human samples.

Three patients with PPP and 5 healthy controls were recruited for this study. Punch biopsies (4 mm) were taken from lesional and nonlesional skin of patients with PPP, and healthy control skin.

### Single-cell sequencing.

Biopsies were bisected before being enzymatically digested in either 0.25% Trypsin-EDTA (Gibco, Thermo Fisher Scientific) with 10 U/mL DNase I (Thermo Fisher Scientific) for 1 hour at 37°C, quenched with FBS (Atlanta Biologicals), or 0.2% Collagenase II (Life Technologies) and 0.2% Collagenase V (MilliporeSigma), with 10U/mL DNase I in plain medium for 1.5 hours at 37°C with rotation. The resulting cell suspensions were filtered through 70 μm cell strainers twice and resuspended in PBS containing 0.04% BSA. Dermal and epidermal cells were combined in a 2:1 ratio. Cell suspensions from tissue were submitted for scRNA-Seq, respectively; libraries were constructed by the University of Michigan Advanced Genomics Core on the 10X Genomics Chromium system with chemistry v2 and v3 and sequenced on the Illumina NovaSeq 6000 sequencer to generate 150 bp paired-end reads.

Data processing, including quality control, read alignment (hg38), and gene quantification, was conducted using the 10X Cell Ranger. The samples were merged into a single expression matrix using the CellRanger aggr pipeline. The R package Seurat (v5.0.2) (37) was used to cluster the cells in the merged matrix. Cells with less than 500 transcripts or 100 genes, or more than 10% of mitochondrial expression, were first filtered out as low-quality cells. SoupX was utilized to remove ambient RNA reads. Doublets were detected and removed using scDblfinder (38). The NormalizeData function was used to normalize the expression level for each cell with default parameters. Samples were batch-corrected using Harmony.

The subclusters were annotated by the marker genes for the subclusters with the canonical subcluster signature genes. IPA was applied to the differentially expressed genes of each cell type to determine the canonical pathways and the potential upstream regulators using IPA. The upstream regulators with an activation *z* score ≥ 2 were considered significantly activated. The module scores were calculated using the AddModuleScore function on the genes induced by the intended cytokine from bulk RNA-Seq analysis. The cytokine-induced genes activating keratinocytes in vitro were obtained from Billi et al. (17) and the “AddModuleScore” function was used to calculate module scores.

### Cell-cell interaction.

After scRNA-Seq data from lesional, nonlesional, and healthy control samples were processed and clustered using the Seurat R package (v4.2.0), each cell was annotated by canonical marker expression into major cell types (e.g., keratinocytes, FBs, lymphocytes, myeloid cells, Schwann cells, melanocytes, smooth muscle, endothelial cells, and mast cells).

Communication networks were inferred separately for each sample group (lesional, nonlesional, healthy) using the CellChat R package (v1.1.3). For each condition, a CellChat object was created from the normalized, log-transformed expression matrix and the corresponding cell-type labels. The built-in human ligand-receptor database was employed, and only ligand-receptor pairs expressed in at least 10% of the cells within a given cluster were retained. Overexpressed genes and interactions were identified, and communication probabilities were computed and filtered to remove low-confidence links (*P* < 0.05). Pathway-level communication probabilities were then aggregated to obtain overall interaction strengths between each sender-receiver cell type pair.

Interaction strengths for all signaling pathways were visualized as heatmaps with cell types arranged on both axes and a color scale representing relative communication weight. Bar plots of total outgoing signal strength per sender cell type were overlaid atop each heatmap to facilitate intercondition comparison.

To focus on the IL-17 signaling pathway, the corresponding subnetwork was extracted from the aggregated communication object. Network centrality measures (sender, receiver, mediator, influencer) were computed for cells in each condition, and the IL-17 signaling network was displayed in a circular layout highlighting regTh17 cells as primary senders to supraspinous keratinocyte receivers.

Finally, expression distributions of key IL-17 pathway ligands (*IL17A*, *IL17F*) and receptors (*IL17RA*, *IL17RC*) were depicted as violin plots across all annotated cell types. Font sizes for axis labels and titles were standardized to ensure legibility. All figures were produced using ggplot2 (v3.4.2) and CellChat’s built-in visualization functions.

### Immunohistochemistry.

Paraffin-embedded PPP (*n* = 3) and healthy skin (*n* = 3) sections were heated at 60°C for 30 minutes, deparaffinized, and rehydrated. Slides were placed in pH 9.0 antigen-retrieval buffer and heated at 125°C for 30 seconds in a pressure cooker. After cooling, sections were treated with 3% H_2_O_2_ for 5 minutes, blocked with 10% goat serum for 30 minutes, and incubated overnight at 4°C with primary antibodies against TNFSF4 (Thermo Fisher Scientific, PA5-116057; rabbit; 2 μg/mL), TNFRSF4 (Thermo Fisher Scientific, BS-2685R; rabbit; 2 μg/mL), TNFSF18 (Thermo Fisher Scientific, PA5-80168; rabbit; 2 μg/mL), TNFRSF18 (Cell Signaling Technology, 10419S; rabbit; 1:50), and FOXP3 (Thermo Fisher Scientific, 13-4777-82; mouse; 10 μg/mL). Slides were then washed, incubated with HRP-conjugated secondary antibody for 30 minutes, and developed with DAB substrate.

### Immunofluorescence.

Paraffin-embedded PPP (*n* = 3) and healthy skin (*n* = 3) sections were heated at 60°C for 30 minutes, deparaffinized, and rehydrated. Slides were placed in pH 9.0 antigen-retrieval buffer and heated at 125°C for 30 seconds in a pressure cooker. After cooling, sections were blocked with 10% donkey serum for 30 minutes and incubated overnight at 4°C with one of the following antibody combinations: IL-17A (LifeSpan Biosciences, LS-C104427; goat; 5 μg/mL), FOXP3 (Thermo Fisher Scientific, 13-4777-82; mouse; 10 μg/mL), or IL-26 (Abcam, AB224198; rabbit; 1:500) together with FOXP3 (Thermo Fisher Scientific, 13-4777-82; mouse; 10 μg/mL). Slides were then washed, incubated with fluorescently conjugated secondary antibody for 30 minutes, washed again, and mounted in fluorescence mounting medium.

### Pseudotime trajectory.

Pseudotime trajectory was constructed using the R package Monocle (39). The raw count data from the Seurat analysis were processed and normalized using the estimateSizeFactors and estimateDispersions functions with default settings. Genes with an average expression exceeding 0.5 and present in at least 10 cells were selected for further investigation. Variable genes were identified using the differentialGeneTest function, modeling against the T cell subclusters. Cell ordering was performed with the orderCells function, and the trajectory was generated using the reduceDimension function with default parameters. A heatmap displaying gene expression along pseudotime was created using the plot_pseudotime_heatmap function. IPA was used to determine the upstream regulators for the DEGs.

### Spatial sequencing library preparation.

Formalin-fixed, paraffin-embedded punch biopsies from patients with PPP (lesional and matched nonlesional skin) and healthy controls were processed on a Leica ASP 300S (15-minute stations), cored, and recombined into a single 10 × 22 mm paraffin block, and they were sectioned at 5 μm onto CosMx Spatial Molecular Imager slides (NanoString), following the manufacturer’s instructions.

### Spatial sequencing analysis.

PPP sample (*n* = 1) for NanoString CosMx analysis were prepared using a 1,000-plex panel. The process involved hybridizing the samples with specific probes and staining them with cell markers. All samples were simultaneously loaded onto the CosMx SMI for imaging. During imaging, branched fluorescent probes were applied to the samples, enhancing signal amplification above the background noise. Flat files (exprMat_file.csv.gz, fov_positions_file.csv.gz, metadata_file.csv.gz, polygons.csv.gz, tx_file.csv.gz) were exported from AtoMX v1.3.2, the Spatial Informatics platform of Nanostring. During preprocessing, cells with fewer than 20 counts, negative probes (feature names starting with “Neg”), and system probes (feature names starting with “System”) were removed from analysis. Cell type annotations were performed using the R package InsituType v1.0.0 in a supervised way, utilizing a reference profile from PPP scRNA-Seq dataset.

With the R package Giotto v4.1.0, a Giotto object was created based on the flat files. Information including the gene expression matrix, cell polygons, and spatial locations of transcripts for each field of view (FOV), were incorporated into a sub-Giotto object with the Giotto function “createGiottoObjectSubcellular.” The sub-Giotto objects of all FOVs were then merged to a comprehensive Giotto object using the Giotto function “joinGiottoObjects.” Insitu plots showing the distribution of selected features overlapped with cells were generated using the Giotto function “spatInSituPlotPoints.” Each dot in a spatial image represents a single transcript detection event; clustering of dots within the boundaries of a segmented cell corresponds to multicopy expression in that cell.

### Neighborhood enrichment analysis.

To quantify local cellular neighborhoods, we extracted per-cell spatial coordinates from our merged Giotto object (Giotto v4.1.0), mapped each cell’s subcluster from the CosMx metadata (metadata_file.csv.gz), and computed pairwise Euclidean distances between all cells within each FOV using base R functions. For each cell, we then identified its 150 nearest neighbors (*k* = 150) and tabulated the counts of neighbor cell types. These per-FOV, per-cell k-nearest neighbors (k-NN) [which refers to the process of finding, for each individual cell, the k closest cells (neighbors)] matrices were saved as sparse matrices and subsequently aggregated across FOVs.

Within each cell type “sender” group, neighbor counts were summed and normalized by the total number of potential neighbors of each “receiver” cell type, yielding a percentage enrichment matrix with rows summing to 100. Cell types with fewer than 20 total cells were excluded to avoid low-frequency artifacts. The resulting neighborhood-enrichment tables (1 for each k) were exported as CSV files and visualized as heatmaps in R using ComplexHeatmap (v2.12.0) with a blue-white-red color ramp (circlize v0.4.15), hierarchical clustering of both axes, and standardized font sizes for row and column labels.

### Per-cell gene expression.

Raw spatial transcriptomics data generated on the CosMx platform were processed and summarized at the cell type level using the Giotto R package (v1.4.0). First, a Giotto object was loaded from the preprocessed output directory, and the raw RNA count matrix (genes × cells) was extracted. Spatial coordinates for each cell were retrieved for downstream spatial analyses but are not directly involved in the expression summarization. Cell metadata including unique cell identifiers, and assigned cell type annotations were accessed via Giotto’s internal data tables.

A panel of 8 genes of interest (*CD3E*, *CD4*, *CTLA4*, *FOXP3*, *IL17A*, *IL17F*, *IL26*, *IL36G*) was defined. For each gene we computed the mean transcript count per cell, the median transcript counts per cell, and the total transcript count across all cells of that type. To visualize cell type–specific expression patterns, bar plots of mean transcript counts were generated for each gene across all annotated cell types.

### Spatial proximity analysis.

To assess spatial interactions between immune cell subsets and IL-36G^+^ keratinocytes, spatial coordinates and raw transcript counts were extracted from the CosMx spatial transcriptomics dataset. Cells expressing IL-36G were identified as IL-36G^+^ keratinocytes. Immune cells were classified into marker-defined populations based on expression of key transcripts: Treg (*CD4^+^FOXP3^+^IL-2RA^+^*), Th17 (*CD4^+^IL-17A^+^RORC^+^*), regTh17 (*IL-17F^+^IL-26^+^FOXP3^+^*), and NKT (*CD8A^+^NKG7^+^KLRD1^+^*). For each immune cell, the Euclidean distance to the nearest IL-36G^+^ keratinocyte was calculated. Distances were stratified into “Near” (<20 μm) and “Far” (≥20 μm) groups. The proportion of each immune subset falling into each distance category was visualized using grouped bar plots. Statistical comparisons were performed using the Wilcoxon rank-sum test.

### Statistics.

The Wilcoxon rank-sum test (2-sided) was utilized for analyzing marker genes and differential expression in scRNA-Seq data. IHC and immunofluorescence experiments were conducted using commercially sourced antibodies, following the specified conditions mentioned earlier, and incorporating appropriate isotype controls as negative controls.

### Study approval.

The study was approved by the University of Michigan IRB HUM00174864, and all patients provided written informed consent. The study was conducted according to the Declaration of Helsinki principles.

### Data availability.

The RNA-Seq data discussed in this publication are available in the NCBI’s Gene Expression Omnibus database (accession no. GSE304122). The CosMx spatial transcriptomics data supporting the findings of this study are available upon request. Values for all data points in graphs are reported in the Supporting Data Values file.

## Author contributions

THD and JEG designed the research studies and wrote the manuscript. THD analyzed the data. RB, HZ, and XX conducted experiments and acquired data. MGK, MR, JF, RJ, OP, PWH, MN, EX, MG, ACB, JMK, RLM, OU, and LCT contributed reagents/materials and edited the manuscript.

## Supplementary Material

Supplemental data

Supporting data values

## Figures and Tables

**Table 1:**
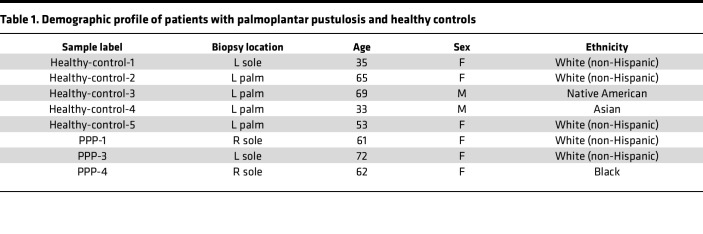
Demographic profile of patients with palmoplantar pustulosis and healthy controls

## Version 1 09/23/2025

Electronic publication
