# Supplementary material for: Th17 cells with regulatory phenotype are the main IL-17F and IL-26 producers in palmoplantar pustulosis
Source: JCI Insight. 2025 Sep 23;10(18):e193038. doi: 10.1172/jci.insight.193038 (PMC12487861; doi:10.1172/jci.insight.193038)
Supplement: Supplemental data [file jciinsight-10-193038-s224.pdf]

Supplementary Fig. 1

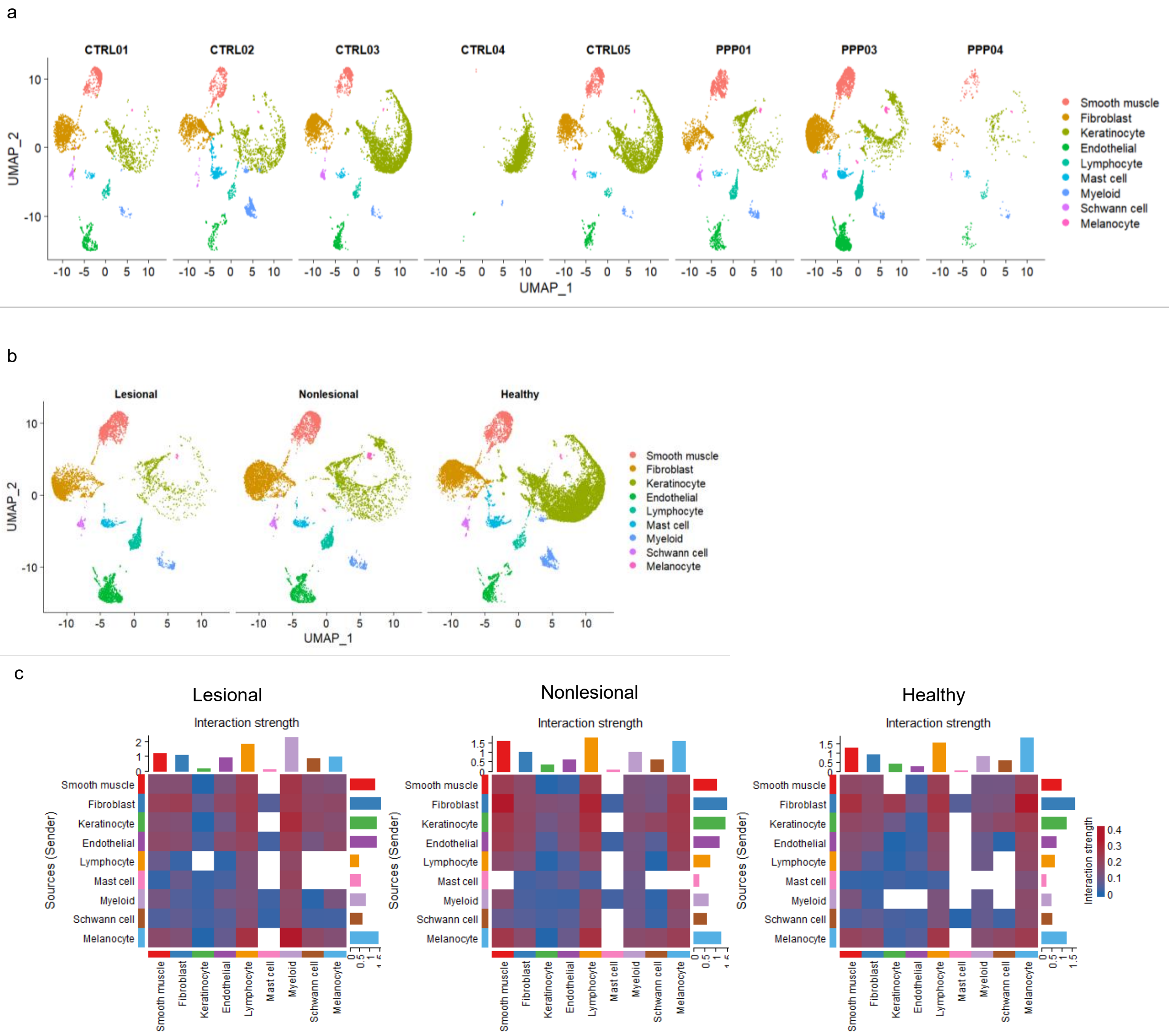

Supplementary Fig. 2

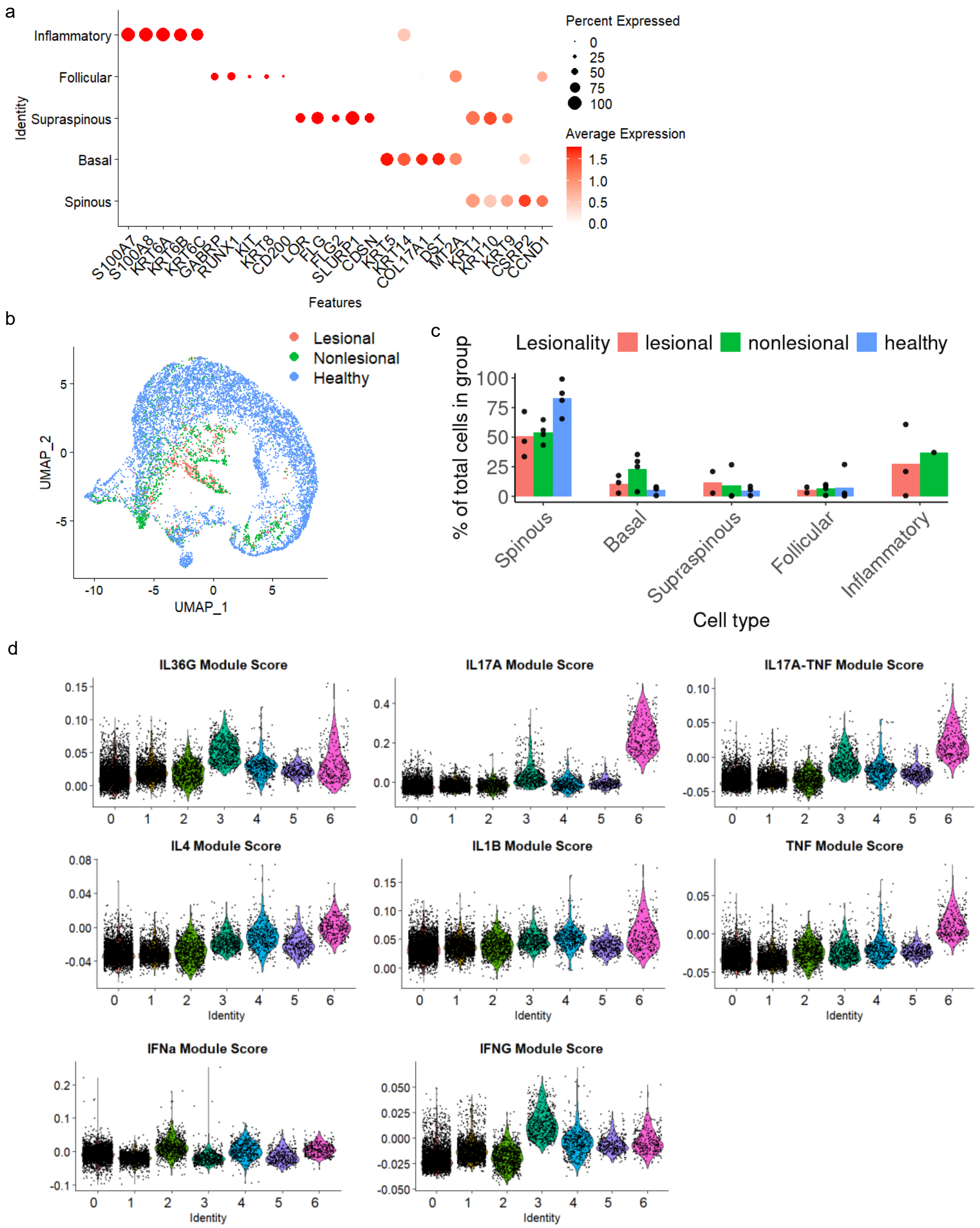

Supplementary Fig. 3

a

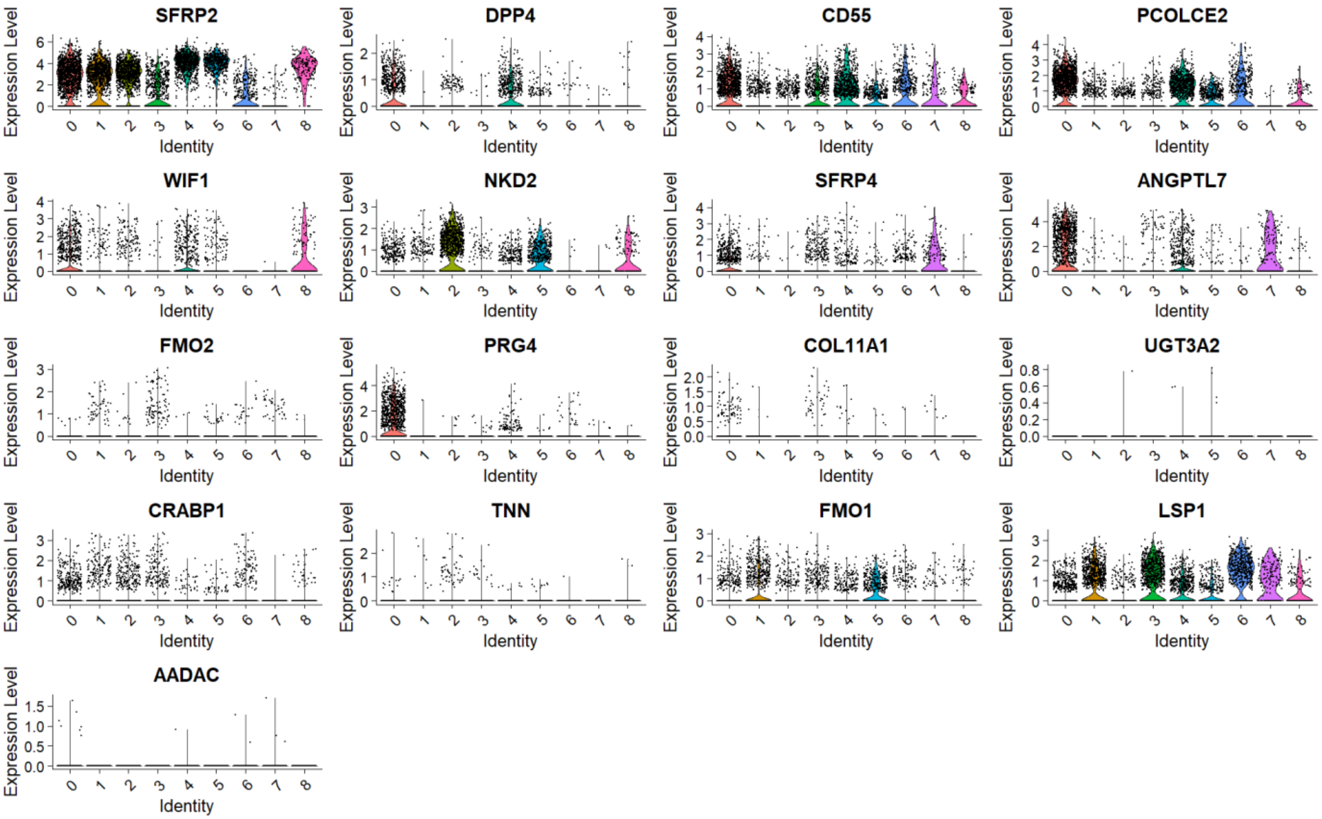

b

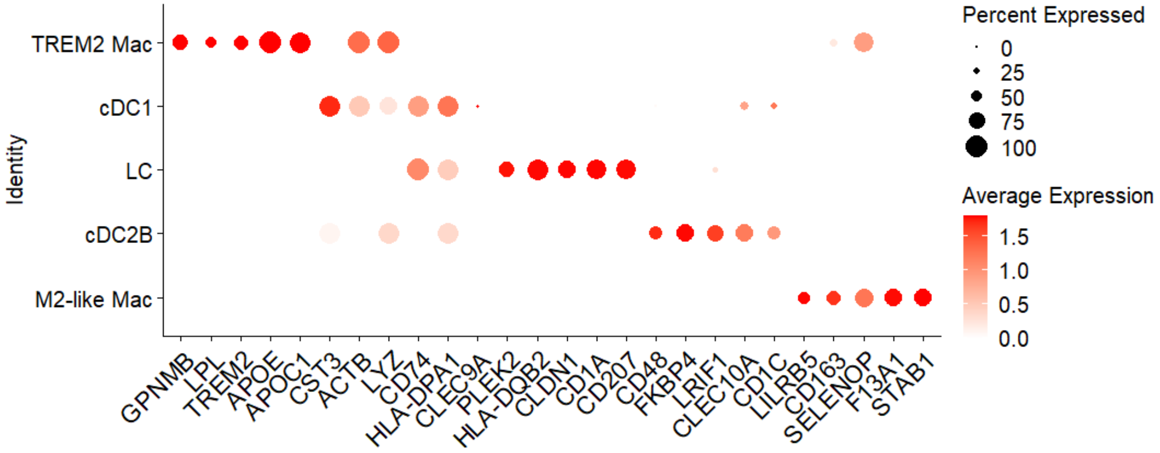

Supplementary Fig. 4

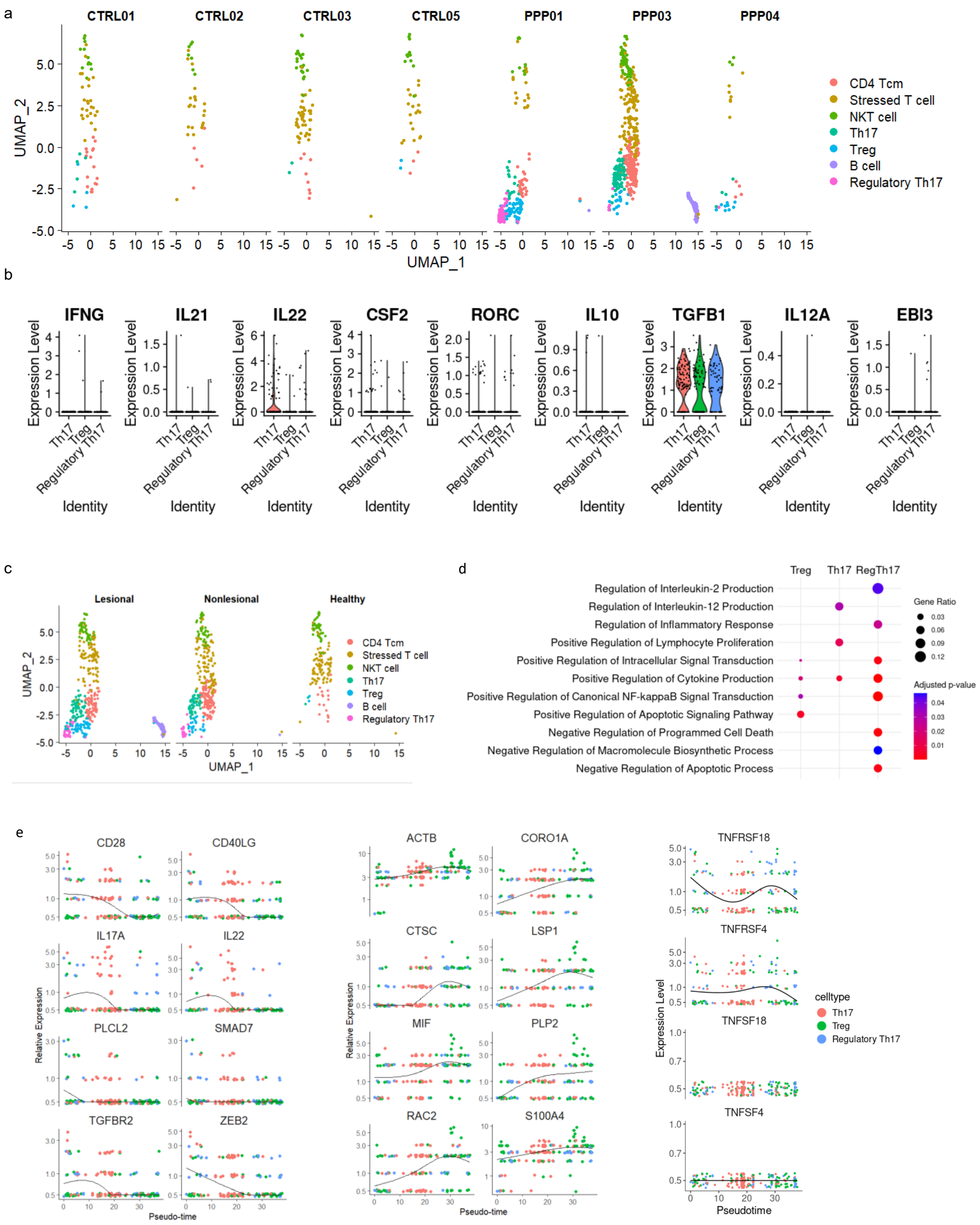

Supplementary Fig. 4

f

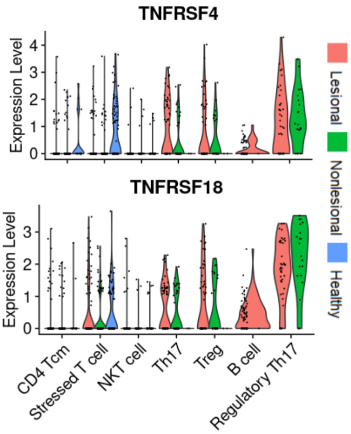

g

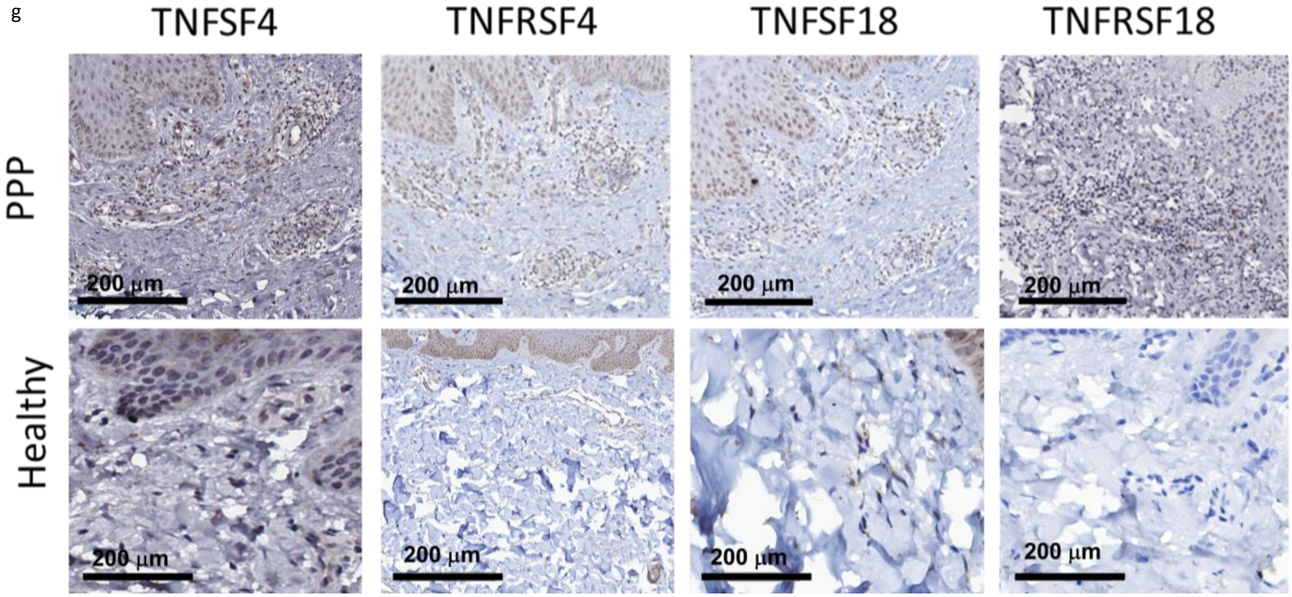

Supplementary Fig. 5

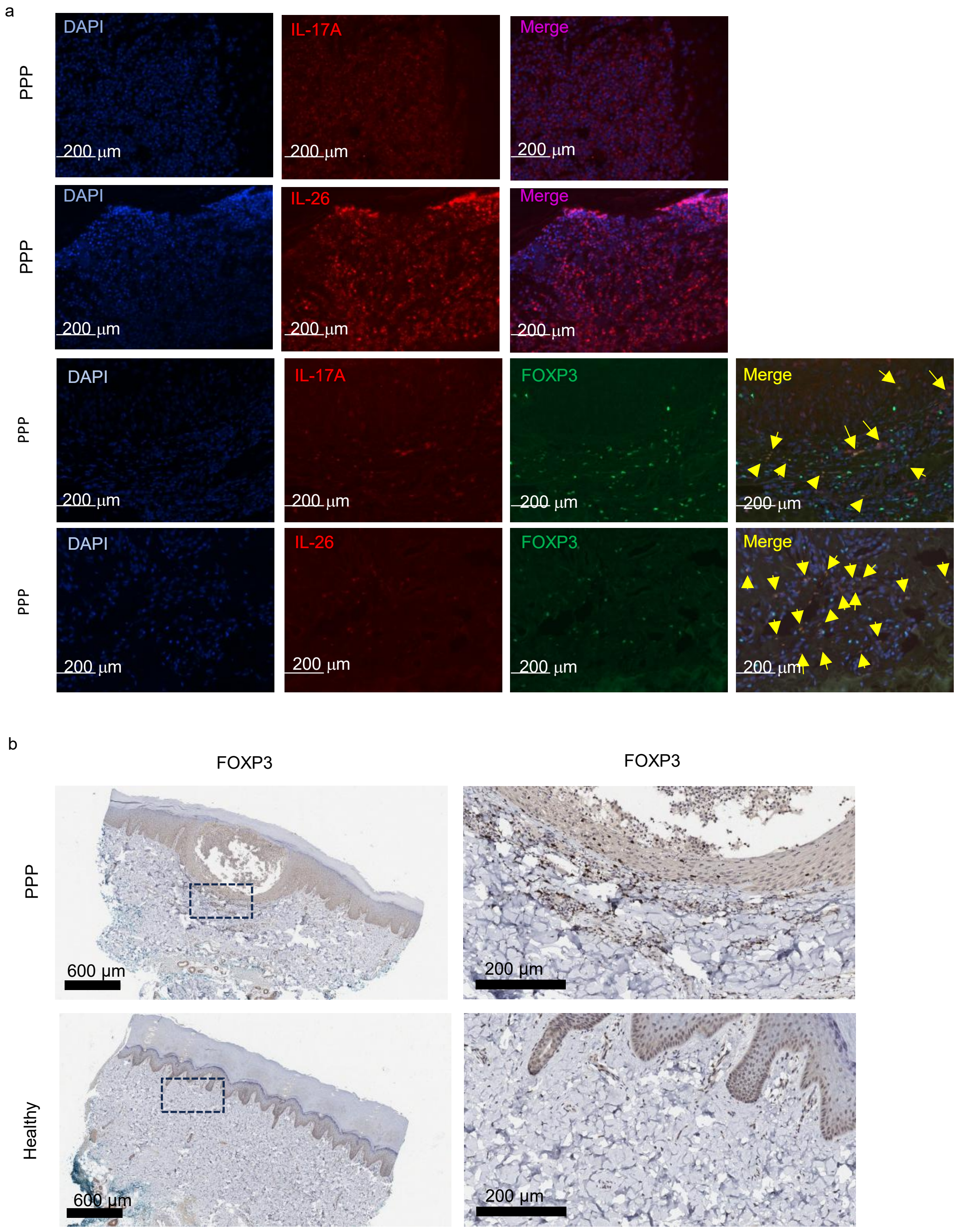

Supplementary Fig. 6

Nonlesional

a

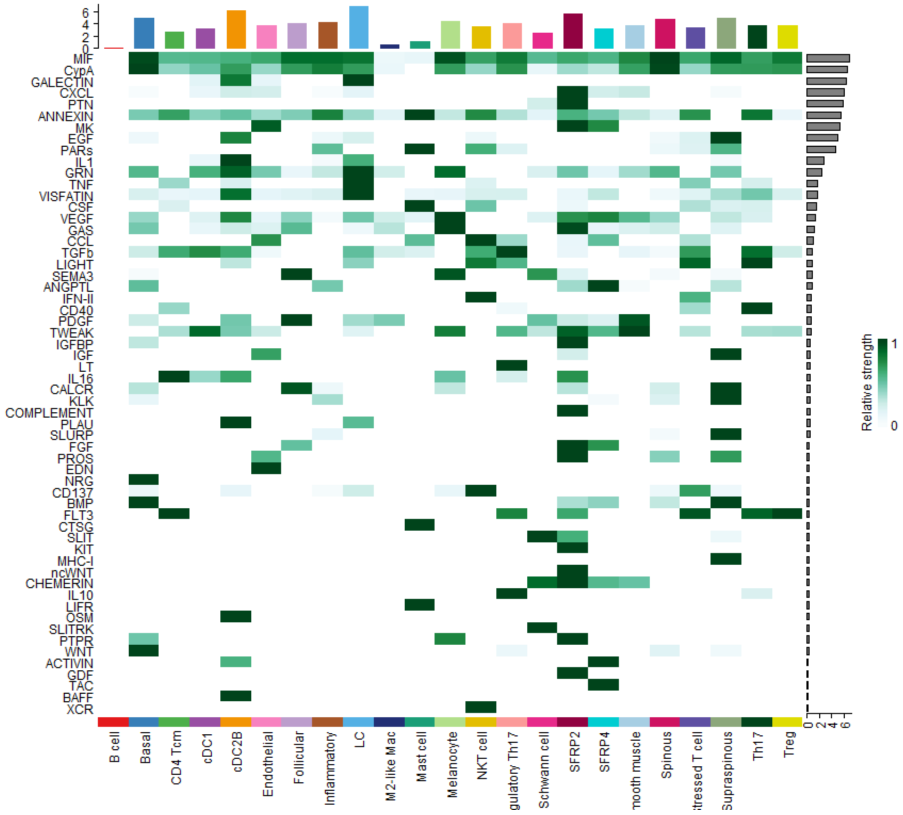

Healthy

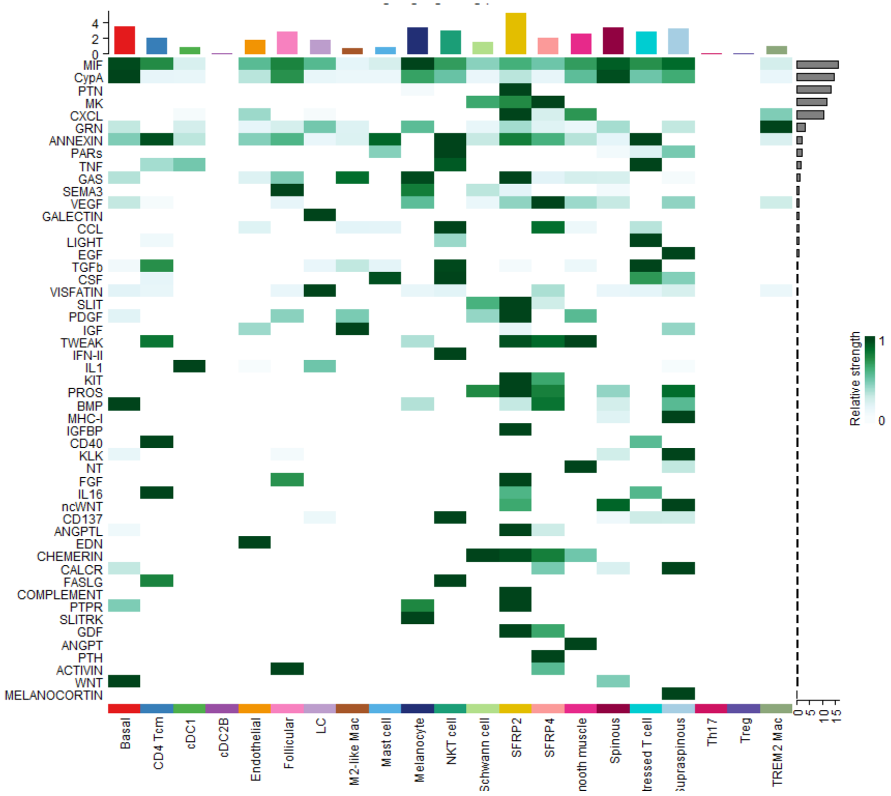

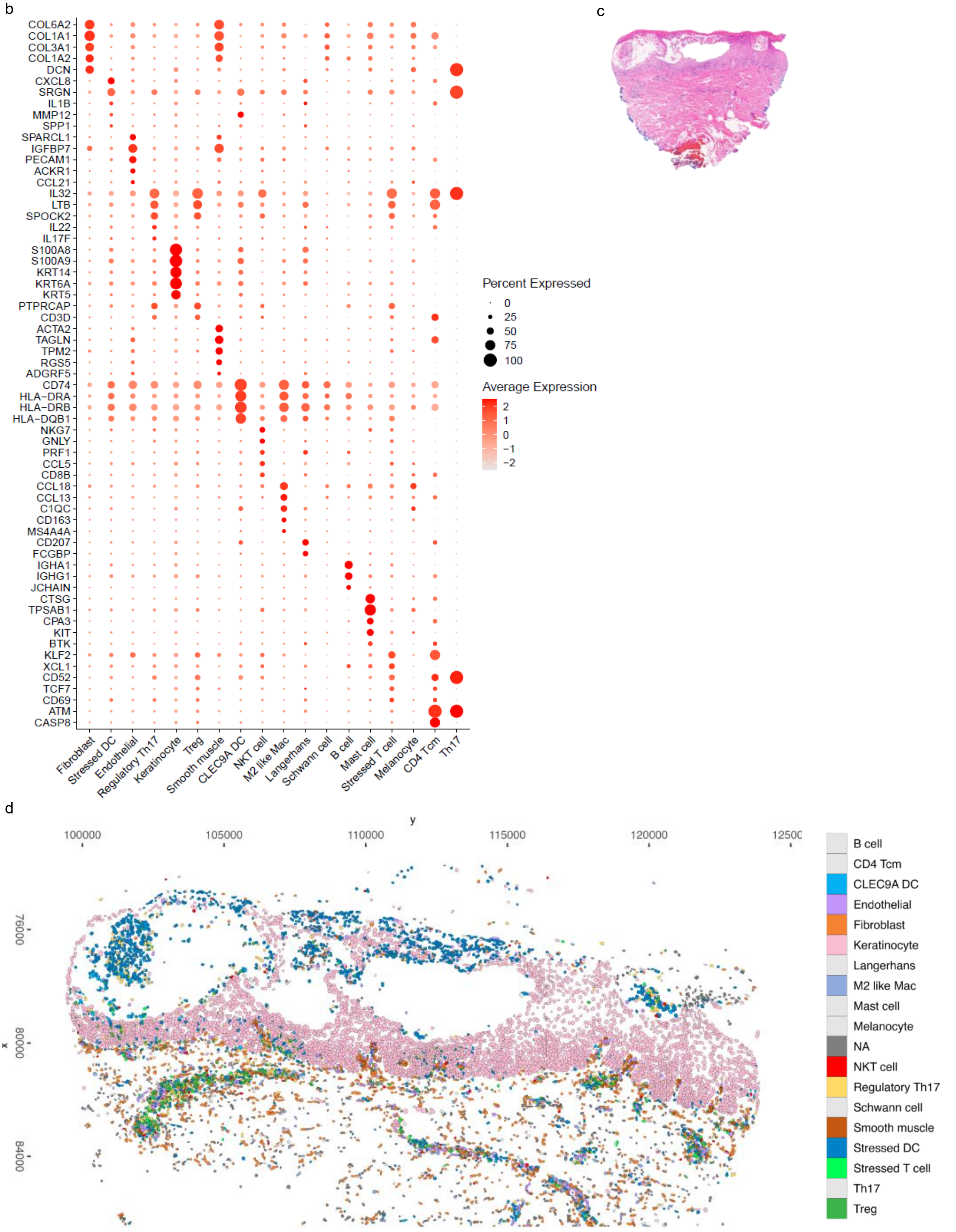
b
COL6A2
COL1A1
COL3A1
COL1A2
DCN
CXCL8
SRGN
IL1B
MMP12
SPP1
SPARCL1
IGFBP7
PECAM1
ACKR1
CCL21
IL32
LTB
SPOCK2
IL22
IL17F
S100A8
S100A9
KRT14
KRT6A
KRT5
PTPRCAP
CD3D
ACTA2
TAGLN
TPM2
RGS5
ADGRF5
CD74
HLA−DRA
HLA−DRB
HLA−DQB1
NKG7
GNLY
PRF1
CCL5
CD8B
CCL18
CCL13
C1QC
CD163
MS4A4A
CD207
FCGBP
IGHA1
IGHG1
JCHAIN
CTSG
TPSAB1
CPA3
KIT
BTK
KLF2
XCL1
CD52
TCF7
CD69
ATM
CASP8
Fibroblast
Stressed DC
Endothelial
Regulatory Th17
Keratinocyte
Treg
Smooth muscle
CLEC9A DC
NKT cell
M2 like Mac
Langerhans
Schwann cell
B cell
Mast cell
Stressed T cell
Melanocyte
CD4 Tcm
Th17
Percent Expressed
0
25
50
75
100
Average Expression
2
1
0
−1
−2
c
d
y
100000
105000
110000
115000
120000
12500
x
76000
80000
84000
B cell
CD4 Tcm
CLEC9A DC
Endothelial
Fibroblast
Keratinocyte
Langerhans
M2 like Mac
Mast cell
Melanocyte
NA
NKT cell
Regulatory Th17
Schwann cell
Smooth muscle
Stressed DC
Stressed T cell
Th17
Treg

Supplementary Fig. 6

e

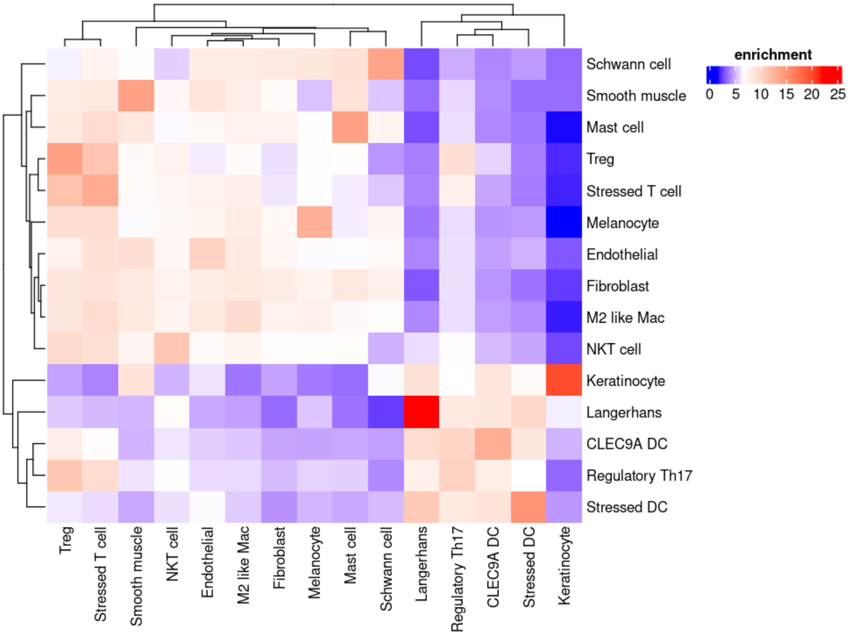

f

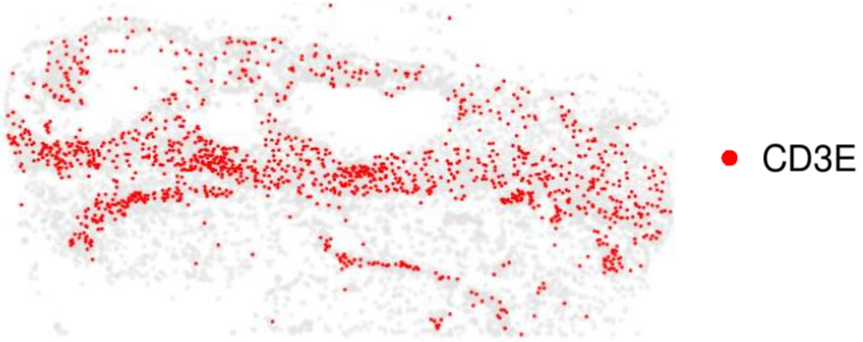

g

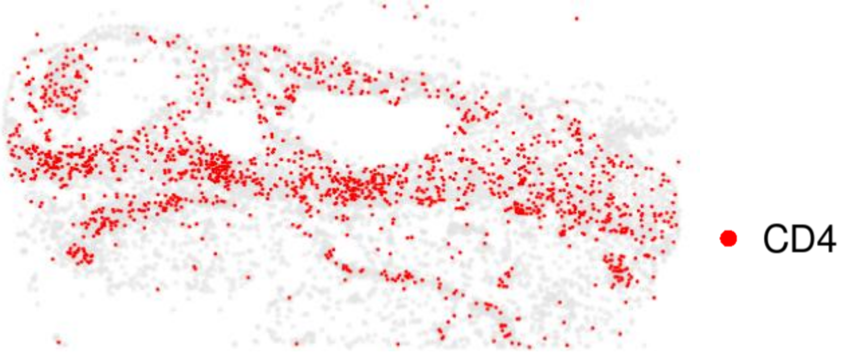

h

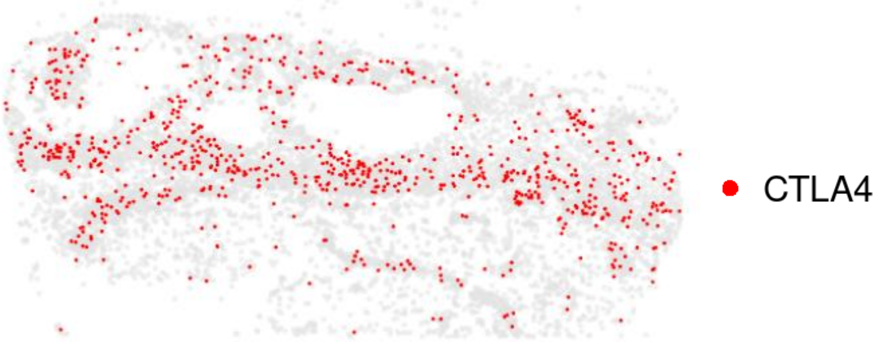

i

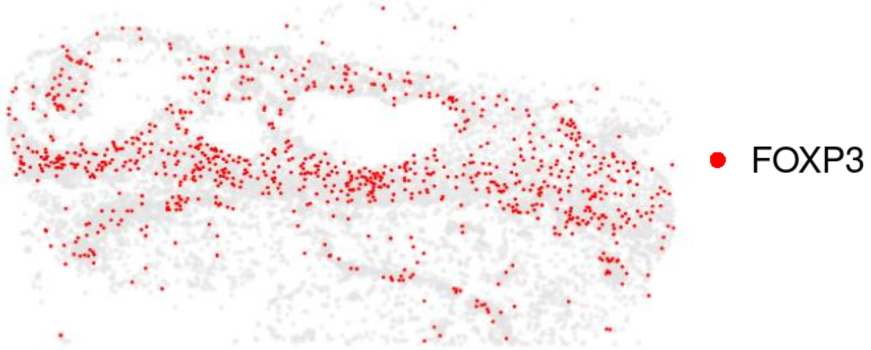

j

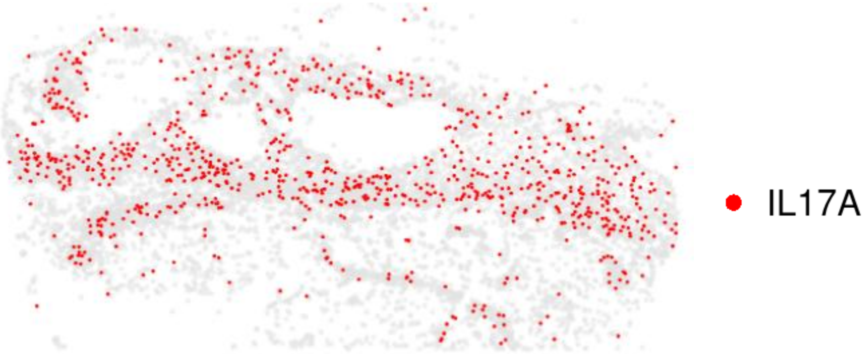

k

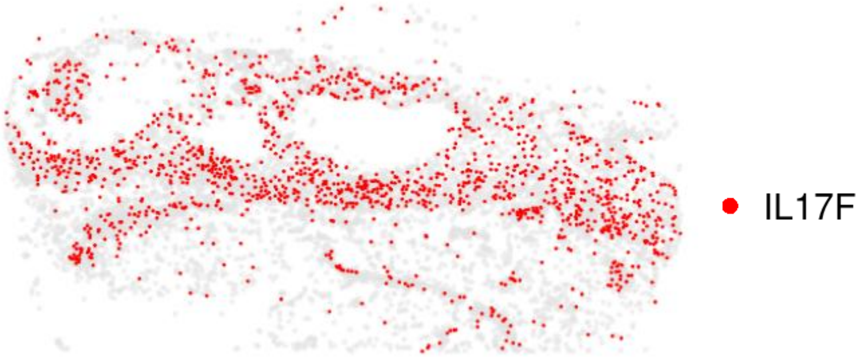

l

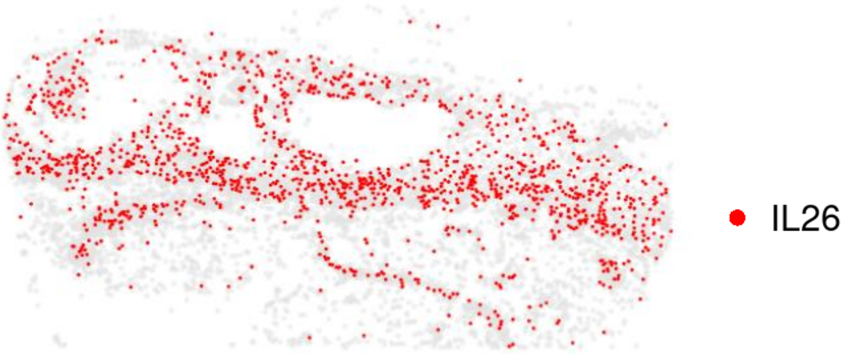

m

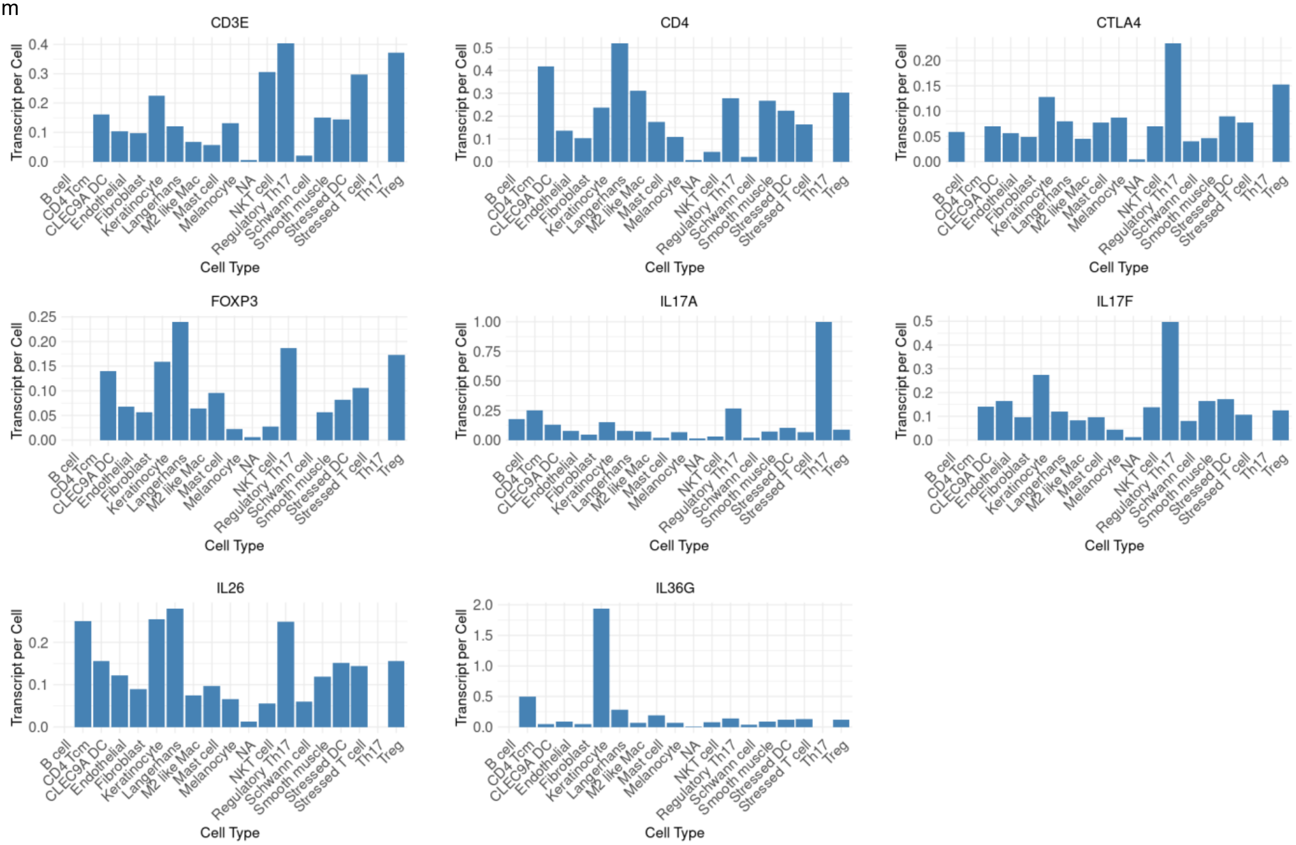
CD3E
CD4
CTLA4
FOXP3
IL17A
IL17F
IL26
IL36G
Transcript per Cell
Cell Type
B cell
CD4 Tcm
CLEC9A DC
Endothelial
Fibroblast
Keratinocyte
Langerhans
M2 like Mac
Mast cell
Melanocyte
NA
NKT cell
Regulatory Th17
Schwann cell
Smooth muscle
Stressed DC
Stressed T cell
Th17
Treg

n

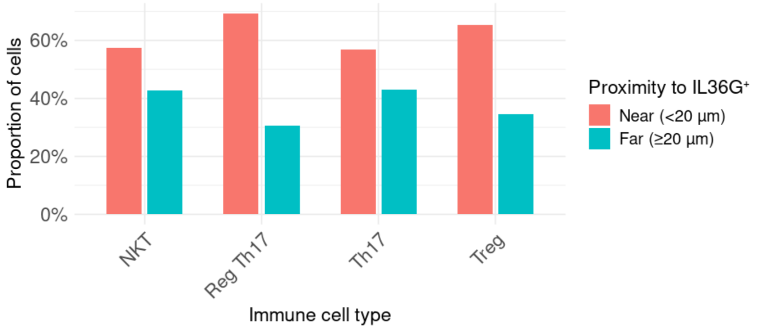
Proportion of cells
60%
40%
20%
0%
NKT
Reg Th17
Th17
Treg
Immune cell type
Proximity to IL36G⁺
Near (<20 μm)
Far (≥20 μm)
